# Hypoxia attenuates the proinflammatory response in colon cancer cells by regulating IκB

**DOI:** 10.18632/oncotarget.3961

**Published:** 2015-04-29

**Authors:** Kamila Müller-Edenborn, Karolin Léger, Jesus F. Glaus Garzon, Carole Oertli, Ali Mirsaidi, Peter J. Richards, Hubert Rehrauer, Patrick Spielmann, David Hoogewijs, Lubor Borsig, Michael O. Hottiger, Roland H. Wenger

**Affiliations:** ^1^ Institute of Physiology, University of Zurich, Zurich, Switzerland; ^2^ Zurich Center for Integrative Human Physiology (ZIHP), University of Zurich, Zurich, Switzerland; ^3^ Institute of Veterinary Biochemistry and Molecular Biology, University of Zurich, Zurich, Switzerland; ^4^ Center for Applied Biotechnology and Molecular Medicine, University of Zurich, Zurich, Switzerland; ^5^ Functional Genomics Center, University of Zurich, Zurich, Switzerland

**Keywords:** inflammatory bowel disease, lipopolysaccharide, NF-κB, tissue oxygenation, tumor hypoxia

## Abstract

Two main features common to all solid tumors are tissue hypoxia and inflammation, both of which cause tumor progression, metastasis, therapy resistance and increased mortality. Chronic inflammation is associated with increased cancer risk, as demonstrated for inflammatory bowel disease patients developing colon cancer. However, the interplay between hypoxia and inflammation on the molecular level remains to be elucidated. We found that MC-38 mouse colon cancer cells contain functional hypoxic (HIF-1α) and inflammatory (p65/RelA) signaling pathways. In contrast to cells of the myeloid lineage, HIF-1α levels remained unaffected in MC-38 cells treated with LPS, and hypoxia failed to induce NF-κB. A similar regulation of canonical HIF and NF-κB target genes confirmed these results. RNA deep sequencing of HIF-1α and p65/RelA knock-down cells revealed that a surprisingly large fraction of HIF target genes required p65/RelA for hypoxic regulation and a number of p65/RelA target genes required HIF-1α for proinflammatory regulation, respectively. Hypoxia attenuated the inflammatory response to LPS by inhibiting nuclear translocation of p65/RelA independently of HIF-1α, which was associated with enhanced IκBα levels and decreased IKKβ phosphorylation. These data demonstrate that the interaction between hypoxic and inflammatory signaling pathways needs to be considered when designing cancer therapies targeting HIF or NF-κB.

## INTRODUCTION

The growth of solid tumors is associated with insufficient oxygen supply (hypoxia) and accompanied by leukocyte infiltration (inflammation), both of which further affect the metabolism of cancer cells, thereby regulating proliferation, metastasis and therapy efficacy [1]. Hypoxia-inducible factor (HIF) is the master regulator of the hypoxic response in cancer cells [2, 3]. HIFs are heterodimeric transcription factors consisting of one of three different oxygen-sensitive HIFα subunits and a common constitutive HIFα subunit. A family of prolyl-4-hydroxylase domain (PHD) enzymes covalently modifies two proline residues within the oxygen-dependent degradation (ODD) domain of HIFα subunits. The PHD family comprises three members called PHD1, PHD2 and PHD3. Upon hydroxylation under normoxic conditions, HIFα is bound by the von Hippel-Lindau (VHL) tumor suppressor protein and targeted for proteasomal destruction. The resulting high turnover rate of HIFα subunits allows for a very rapid stabilization under hypoxic conditions, affecting the transcriptional activity of several hundred target genes [4]. Therefore, tumor hypoxia and high HIFα levels are associated with poor prognosis of virtually all cancer types [5].

The association between inflammatory bowel disease and colon cancer is one example of how chronic inflammation can result in a pre-disposition to cancer [6]. Correspondingly, anti-inflammatory drugs decrease the incidence of cancer [7]. Invading inflammatory cells represent a remarkable proportion of the total tumor mass and are required for tumor angiogenesis and metastasis [8]. The nuclear factor (NF)-κB is the central transcription factor activated by inflammation. Canonical NF-κB activation is controlled by inhibitor of NF-κB (IκB) kinases (IKK), mainly IKK-κ, needed for phosphorylation-induced degradation of IκB in response to infection and inflammation [9]. Like HIF-1α, NF-κB can be activated in tumors by extrinsic factors (e.g. autoimmune disease) or intrinsic factors (e.g. oncogenes) [7]. In cancer cells, NF-κB regulates the expression of genes involved in several processes that play key roles in tumor progression such as proliferation, migration and apoptosis [10].

Whether or not NF-κB can also be induced by hypoxia is currently unclear. While some groups reported increased NF-κB activation under hypoxic conditions, others could not detect an increased NF-κB DNA-binding activity in hypoxic nuclear extracts, despite strongly induced HIF levels [11-16]. This could be explained by cell-type specific effects as well as by the severity and duration of the hypoxic stimulus which may vary among distinct transcription factors. However, in neutrophils HIF-1 has been suggested to induce NF-κB directly [12]. Moreover, IKK-β has been reported to be a specific PHD1 oxygen sensor target [13], and the ankyrin repeats of p105 NF-κB precursor and IκBα have been shown to be hydroxylated by factor inhibiting HIF (FIH), albeit without any functional consequences [17].

The gene encoding HIF-1α has been suggested to represent a direct NF-κB target [18-22]. So far, TNFα, oxidative stress and bacterial infections have been shown to activate HIF-1α gene transcription via NF-κB. A role for NF-κB in enhancing HIF-dependent tumor progression via inflammatory NF-κB activation has not been investigated yet. To better understand the mutual regulation of hypoxia and inflammation signaling pathways in cancer, we studied their interaction in the mouse colon carcinoma cell line MC-38.

## RESULTS

### The hypoxic response of MC-38 colon cancer cells is mediated by HIF-1

**Figure 1:**
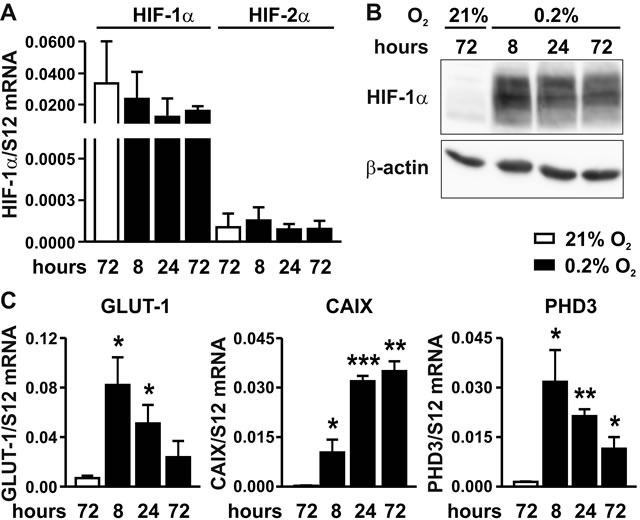
Hypoxic response of MC-38 cells **A.** Time-dependent RT-qPCR quantification of HIF-1α and HIF-2α mRNA levels following exposure to hypoxia as indicated. **B.** Kinetics of hypoxic HIF-1α protein accumulation analysed by immunoblotting of 50 μg total protein extracts. **C.** Kinetics of hypoxic induction of canonical HIF target genes quantified by RT-qPCR of GLUT-1, CAIX and PHD3 mRNA. Shown are mean values + SD of mRNA ratios relative to the constitutively expressed ribosomal protein S12 mRNA levels of *n* = 3 independent experiments; *, *p* < 0.05; **, *p* < 0.01; ***, *p* < 0.001 (Student's *t*-test).

Mouse MC-38 colon adenocarcinoma cells were exposed to normoxia (21% oxygen) or hypoxia (0.2% oxygen) for 8 to 72 hours using conventional two-dimensional (2D) dish culture, and mRNA levels of HIF-1α and HIF-2α were determined by RT-qPCR. Whereas hypoxia did not upregulate HIF-1α mRNA levels (Figure 1A), it robustly induced HIF-1α protein accumulation (Figure 1B). In contrast, only marginal HIF-2α mRNA levels (Figure 1A) and no HIF-2α protein (data not shown) could be detected, suggesting that HIF-1 rather than HIF-2 mediates the hypoxic response in MC-38 cells. No difference in viability and only slightly decreased proliferation rates were observed under hypoxic conditions (data not shown). Transcript levels of the three canonical HIF target genes *Glut1*, *Ca9* and *Phd3* were found to be maximally upregulated by 12, 146 and 23-fold, respectively, with different kinetics (Figure 1C).

### A proinflammatory stimulus activates the NF-κB pathway in MC-38 colon cancer cells

**Figure 2:**
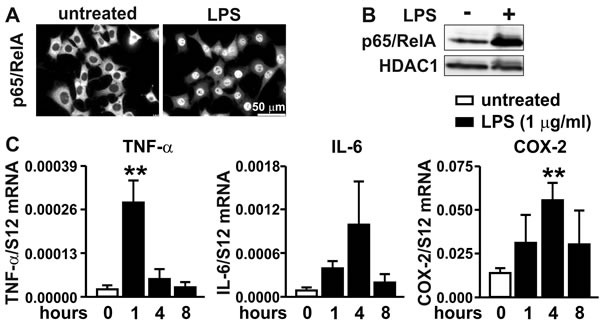
Inflammatory response of MC-38 cells **A.** Immunofluorescence microscopy of p65/RelA in MC-38 cells following treatment with 1 μg/ml LPS for 40 minutes. **B.** Immunoblot detection of p65/RelA and HDAC1 in nuclear extracts derived from MC-38 cells following stimulation with 1 μg/ml LPS for 1 hour. **C.** Kinetics of inflammatory induction (1 μg/ml LPS for the time periods indicated) of canonical NF-κB target genes quantified by RT-qPCR of TNF-α, IL-6 and COX-2 mRNA. Shown are mean values + SD of mRNA ratios relative to the constitutively expressed ribosomal protein S12 mRNA levels of *n* = 3 to 4 independent experiments; **, *p* < 0.01 (Student's t-test).

While the activation of the inflammatory NF-κB pathway by bacterial lipopolysaccharides (LPS) is well established mainly in the myeloid lineage, it is less clear whether all components of this pathway are also active in carcinoma cells. Therefore, we stimulated 2D cultured MC-38 colon cancer cells with LPS and analyzed p65/RelA subcellular localization by immunofluorescence (Figure 2A) and by immunoblotting of nuclear extracts (Figure 2B). LPS induced nuclear translocation of p65/RelA, demonstrating a functional LPS signaling pathway resulting in NF-κB induction. This conclusion was confirmed by a robust transcriptional activation of canonical NF-κB target genes *Tnfa* and *Cox2* which were maximally induced by 13- and 4-fold, respectively, with different kinetics (Figure 2C). Similarly, *Il6* gene expression was upregulated 12-fold by LPS, though not reaching statistical significance (*p* = 0.054).

### NF-κB is not activated by hypoxia in MC-38 colon cancer cells

**Figure 3:**
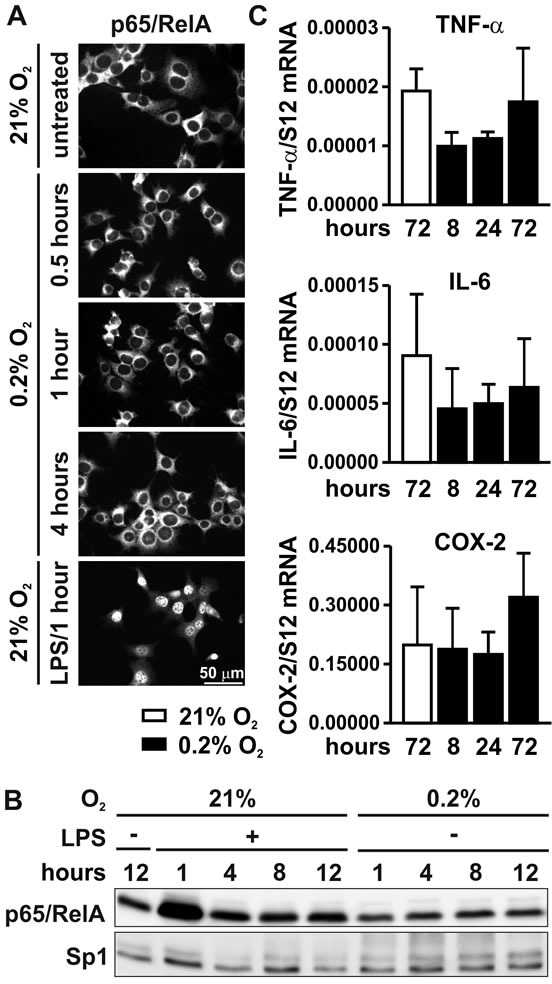
NF-κB signaling under hypoxic conditions **A.** Immunofluorescence microscopy of p65/RelA in MC-38 cells following exposure to 0.2% oxygen for 0.5 to 4 hours. Treatment with 1 μg/ml LPS for 1 hour served as positive control. **B.** Immunoblot detection p65/RelA and the constitutive Sp1 transcription factors in MC-38 nuclear extracts following exposure to 1 μg/ml LPS or 0.2% oxygen for 1 to 12 hours. **C.** RT-qPCR analysis of the regulation of the canonical NF-κB target genes *Tnfa*, *Il6* and *Cox2* following hypoxic exposure.

It has been suggested that hypoxia directly activates the NF-κB pathway by blocking PHD-mediated hydroxylation of IKKβ, at least in HeLa cervical carcinoma cells [13]. However, in contrast to LPS treatment, exposure of MC-38 colon carcinoma cells to hypoxia for 0.5 to 4 hours did not induce nuclear translocation of p65/RelA as shown by immunofluorescence (Figure 3A) and immunoblotting of nuclear extracts (Figure 3B), although HIF-1α proteins levels were stabilized under these conditions (Figure 1B). Consistent with these results, the mRNA levels of the NF-κB target genes *Tnfa*, *Il6* and *Cox2* were not induced in MC-38 cells exposed to hypoxia (Figure 3C). In conclusion, direct hypoxic induction of the NF-κB signaling pathway does not appear to be a general phenomenon in carcinoma cells.

### The *Hif1a* gene is not a transcriptional target of NF-κB in MC-38 colon cancer cells

**Figure 4:**
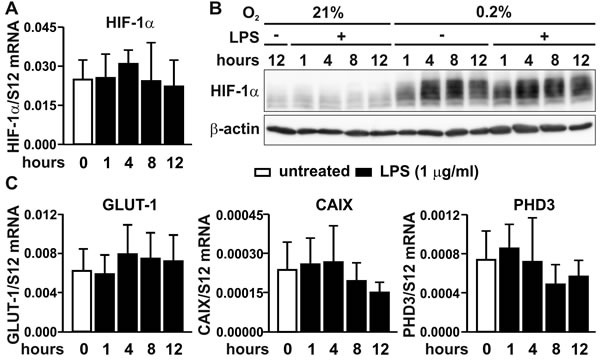
HIF signaling under inflammatory conditions **A.** RT-qPCR analysis of the HIF-1α mRNA response to LPS treatment in MC-38 cells. **B.** Kinetics of the HIF-1α protein response to LPS and/or hypoxia as assessed by immunoblotting of 50 μg total cell extracts. β-Actin served as loading control. **C.** RT-qPCR analysis of the effects of LPS treatment on the canonical HIF targets gene *Glut1*, *Ca9* and *Phd3*.

Several previous reports have demonstrated that NF-κB binds to the promoter of the *Hif1a* gene and activates its transcription in a limited number of cell types [18-21]. However, no significant change in HIF-1α mRNA (Figure 4A) or protein (Figure 4B) levels could be detected in MC-38 cells treated with LPS, regardless of the oxygen concentration. These results were confirmed by the absence of any significant changes in the mRNA levels of the canonical HIF target genes *Glut1*, *Ca9* and *Phd3* (compare Figures 1C and 4C).

### Knock-down of p65/RelA does not affect HIF signaling

**Figure 5:**
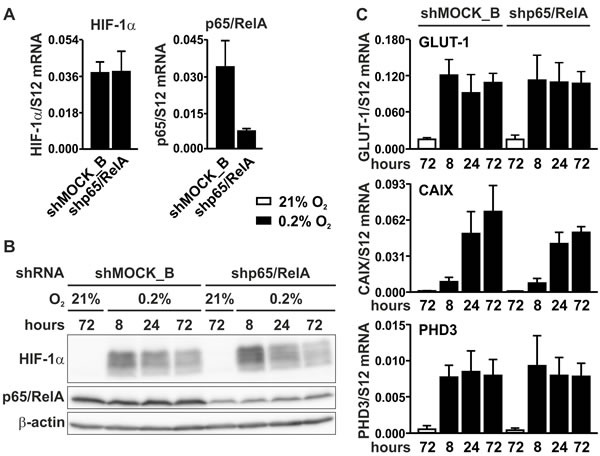
HIF signaling in p65/RelA knock-down cells **A.** RT-qPCR analysis of HIF-1α and p65/RelA mRNA in MC-38 cells stably transfected with shp65/RelA or shMOCK_B negative control constructs. **B.** Immunoblotting of HIF-1α and p65/RelA protein after 8 to 72 hours of hypoxic exposure of shMOCK_B or shp65/RelA MC-38 cells. β-Actin served as loading control. **C.** RT-qPCR analysis of the canonical HIF target genes *Glut1*, *Ca9* and *Phd3* in shMOCK_B or shp65/RelA MC-38 cells cultured under normoxic or hypoxic conditions as indicated.

To investigate whether NF-κB could be involved in basal rather than in conditional HIF-1α regulation, p65/RelA was knocked-down by RNA interference. MC-38 cells were stably transfected with a shRNA construct targeting p65/RelA, leading to an efficient decrease in p65/RelA mRNA (Figure 5A) and protein (Figure 5B) levels. By contrast, HIF-1α mRNA (Figure 5A) and protein (Figure 5B) levels remained unaltered following p65/RelA knockdown. These results were further corroborated by mRNA quantification of the HIF target genes *Glut1*, *Ca9* and *Phd3*, which showed similar profiles in both shMOCK and shp65/RelA treated cells under normoxic as well as hypoxic conditions (Figure 5C).

### Knock-down of HIF-1α does not affect NF-κB signaling

**Figure 6:**
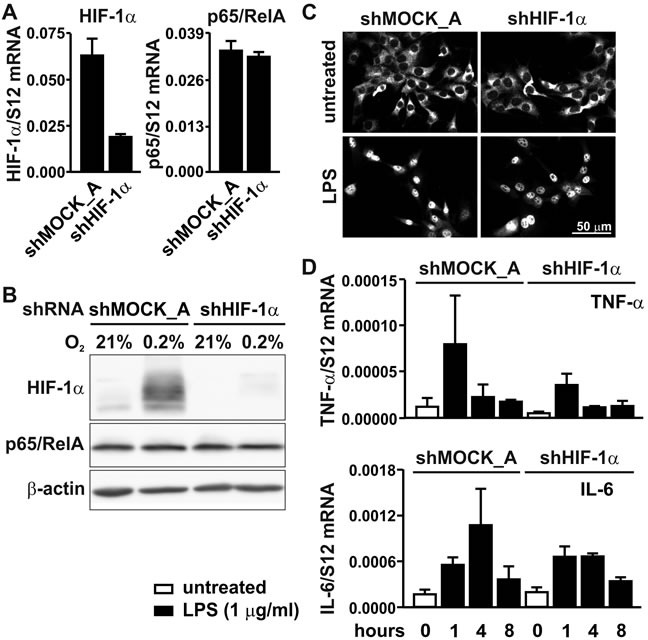
p65/RelA signaling in HIF-1α knock-down cells **A.** RT-qPCR analysis of HIF-1α and p65/RelA mRNA in MC-38 cells stably transfected with shHIF1α or shMOCK_A control constructs. **B.** Immunoblotting of HIF-1α and p65/RelA protein after 8 hours of hypoxic exposure of shMOCK_A or shHIF1α MC-38 cells. β-Actin served as loading control. **C.** Immunofluorescence microscopy of p65/RelA in shMOCK_A or shHIF-1α MC-38 cells treated with 1 μg/ml LPS for 40 minutes. **D.** RT-qPCR analysis of the canonical NF-κB target genes *Tnfa* and *Il6* in shMOCK_A or shHIF-1α MC-38 cells upon treatment with LPS for 1 to 8 hours.

To investigate the converse possibility that HIF-1α is involved in basal rather than conditional NF-κB regulation, HIF-1α was knocked-down by stable shRNA expression. Because another retroviral vector backbone was used, two different shMOCK controls were generated, termed shMOCK_A (Figure 6) and shMOCK_B (Figure 5) for comparison with shHIF-1α and shp65/RelA, respectively. HIF-1α knock-down efficiency was confirmed on the mRNA (Figure 6A) and protein (Figure 6B) levels. However, p65/RelA mRNA levels (Figure 6A) and total p65/RelA protein (Figure 6B) remained unaltered in shHIF-1α treated cells under either normoxic or hypoxic conditions. As shown in Figure 6C, p65/RelA nuclear translocation following LPS treatment also remained unaffected in HIF-1α knock-down MC-38 cells. These results were consistent with the mRNA regulation of the NF-κB target genes *Tnfa* and *Il6*, which showed no significant difference following induction by LPS for 1 to 8 hours (Figure 6D).

### Unexpected cross-talk between hypoxic and inflammatory pathways

In order to obtain a comprehensive picture of the transcriptome regulation by the interaction between hypoxic and inflammatory pathways, MC-38 cells were further characterized by RNA deep sequencing. In order to more closely recapitulate solid tumor conditions, cells were cultured as small (approx. 250 μm diameter) three-dimensional (3D) spheroids in hanging drops. Of note, 3D spheroid conditions appeared to enhance the inflammatory response compared to conventional 2D sub-confluent conditions in tissue culture plates. Following treatment with LPS, the TNF-α mRNA induction rose from 7-fold in 2D to 21-fold in 3D (supplementary Figure S1A). Although we and others previously found hypoxic conditions in the core of larger tumor spheroids [23], HIF-1α remained largely unaffected in the relatively small MC-38 3D spheroid cultures (supplementary Figure S1B).

Stable shHIF-1α, shp65/RelA or shMOCK MC-38 cells were cultured under 3D conditions for 48 hours, exposed to 0.2% oxygen for 8 hours and treated with 1 μg/ml LPS for the last 1 hour of the hypoxic exposure. These dosages and time points were previously determined to be optimal for HIF-1 and NF-κB activation (see also Figures 1 and 2). RNA deep sequencing and subsequent analysis was performed as described in the Materials and Methods section. Thresholds for induction and repression were chosen to be at least 1.5-fold or 0.67-fold, respectively. In the case of cells transfected with shHIF-1α or shp65/RelA, “repression” and “induction” in comparison with shMOCK-transfected cells refers to genes positively and negatively regulated by HIF-1α and/or p65/RelA, respectively (supplementary Table S1).

In summary, for the correct regulation of the majority of the hypoxically induced genes, not only was a functional HIF pathway required (72% of all tested genes required HIF) but also a functional NF-κB pathway (77%). While 59% of the genes induced by the inflammatory stimulus were dependent on NF-κB, only 18% required HIF. Finally, the number of genes induced by LPS under normoxic conditions was reduced by almost 50% under hypoxic conditions, suggesting that hypoxia attenuates the inflammatory response of MC-38 cells.

### Role of HIF-1α in the suppression of proinflammatory gene expression by hypoxia?

**Figure 7:**
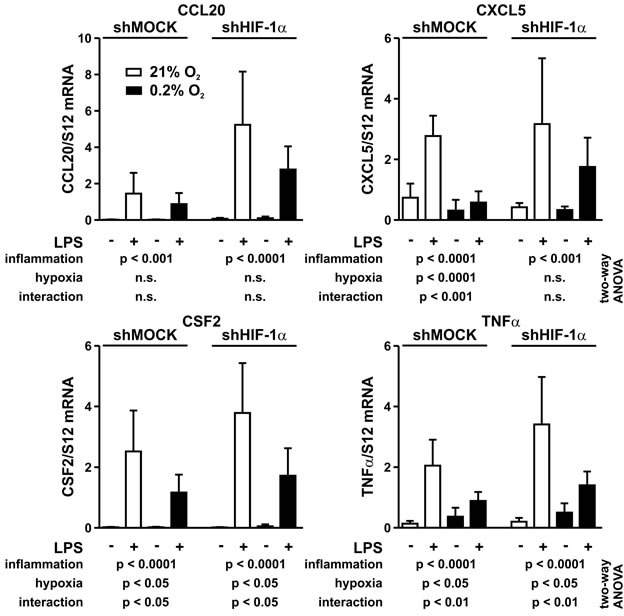
Interaction between inflammatory and hypoxic signaling MC-38 shMock and shHIF-1α cells were exposed to hypoxia (0.2% oxygen) for 8 hours and treated with 1 μg/ml LPS for the last 1 hour of normoxic or hypoxic cell culture. CCL20, CXCL5, CSF2 and TNFα mRNA levels were quantified by RT-qPCR. Shown are mean values + SD of mRNA ratios relative to the constitutively expressed ribosomal protein S12 mRNA levels, normalized to the mean of each experiment (n = 6 independent experiments). Two-way ANOVA was used to assess the significance of the effects of inflammation (LPS) and hypoxia (0.2% oxygen) as well as of the interaction between these two conditions (i.e. the effect of hypoxia on inflammation). *p* values are indicated; n.s., not significant.

A possible explanation for the attenuated inflammatory response under hypoxic conditions might be a HIF-1α dependent inhibition of proinflammatory gene expression. From the hypoxically suppressed proinflammatory genes identified by RNA sequencing, four secreted inflammatory factors (CCL20, CXCL5, CSF2 and TNFα) associated with tumor inflammation were chosen for further validation. Therefore, MC-38 cells were independently exposed to hypoxic (0.2% oxygen) and/or inflammatory (1 μg/ml LPS) conditions, followed by mRNA quantification and data analysis by two-way ANOVA (Figure 7).

All four genes were strongly induced by inflammatory conditions. As expected, this induction is mediated by NF-κB since p65/RelA knock-down blocked their induction by LPS (data not shown). In contrast, hypoxia alone had diverse effects on these genes. While it did not affect CCL20, the mRNA levels of CXCL5, CSF2 and TNFα were significantly altered by hypoxia. HIF-1α knock-down only affected the hypoxic impact on CXCL5 but there were no changes for CCL20, CSF2 and TNFα between shMOCK and shHIF-1α cells.

Importantly, hypoxia significantly affected the inflammatory response of CXCL5, CSF2 and TNFα, while the hypoxic attenuation of the inflammatory CCL20 induction did not quite reach statistical significance. Again, HIF-1α knock-down only prevented the cross-talk between inflammation and hypoxia of CXCL5, but not of CCL20, CSF2 and TNFα (Figure 7), suggesting that HIF is not a major pathway involved in the hypoxic attenuation of proinflammatory gene expression. Indeed, whereas hypoxia blocked the inflammatory induction of 136 out of 277 LPS-induced genes, only 6 and 9 genes, respectively, were negatively regulated by HIF-1α under normoxic and hypoxic conditions (supplementary Table S1).

### Hypoxia impairs LPS-stimulated NF-κB nuclear translocation by increasing IκBα levels

**Figure 8:**
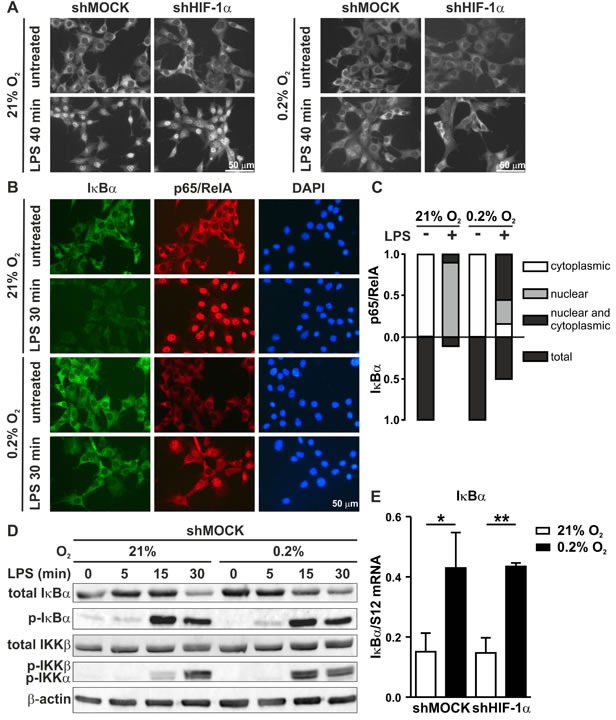
Inverse regulation of NF-κB and IκB under hypoxic conditions **A.** Immunofluorescence microscopy of p65/RelA in shMOCK and shHIF-1α MC-38 cells following exposure to 0.2% oxygen for 8 hours and/or 1 μg/ml LPS for the last 40 minutes before harvesting. **B.** Co-immunofluorescence microscopy of IκBα and p65/RelA in MC-38 cells (without GFP) exposed to 0.2% oxygen for 8 hours and/or 1 μg/ml LPS for the last 30 minutes before harvesting. **C.** For fractional quantification of the results shown in **B.**, at least 200 cells of each experiment were classified according to the subcellular localization of p65/RelA and the expression of IκBα as indicated. **D.** Immunoblot analysis of total IκBα, phosphorylated IκBα, total IKKβ and phosphorylated IKKβ/α in MC-38GFP cells exposed to 0.2% oxygen for 8 hours and 1 μg/ml LPS for the last 5, 15 or 30 minutes as indicated. **E.** RT-qPCR analysis of IκBα mRNA levels in MC-38GFP cells exposed to 8 hours hypoxia and/or 1 hour LPS. Shown are mean values + SD of mRNA ratios relative to the constitutively expressed ribosomal protein S12 mRNA levels, normalized to the mean of each experiment (*n* = 3 independent experiments); *, *p* < 0.05; **, *p* < 0.01 (Student's *t*-test).

Since nuclear translocation is the key event in NF-κB activation, p65/RelA subcellular localization was analyzed by immunofluorescence microscopy of 2D MC-38 cultures. Interestingly, while LPS strongly induced p65/RelA nuclear translocation, this was partially prevented by simultaneous hypoxic exposure in HIF-1α wild-type as well as knock-down cells (Figure 8A), suggesting that the observed block was dependent on hypoxia, but independent of HIF-1α. A similar result was obtained with 3D MC-38 cultures (supplementary Figure S2).

To elucidate the mechanism of impaired p65/RelA nuclear translocation, p65/RelA and IκBα were analyzed by co-immunofluorescence microscopy of MC-38 cells not containing the GFP gene (Figure 8B). Of note, IκBα immunofluorescence intensity appeared to be stronger under hypoxic than normoxic conditions (Figure 8B). A quantitative comparison of the number of cells with nuclear p65/RelA localization following LPS stimulation revealed an inverse association with IκBα. Consistent with the attenuated NF-κB response, more cells were IκBα positive under hypoxic conditions (Figure 8C).

To corroborate these findings and to analyze the underlying mechanisms, both total and phosphorylated IκBα and IKK protein levels were assessed by immunoblotting of MC-38 cells treated as in Figure 8B but with additional time points of LPS stimulation (Figure 8D). Again, total IκBα protein was reproducibly increased by hypoxia alone. Consistently, a faint constitutive IκBα phosphorylation was detectable in unstimulated normoxic but not hypoxic cells. We further found a HIF-independent 3-fold induction of IκBα mRNA levels by exposure to hypoxia alone for 8 hours (Figure 8E). Together, these results suggest that hypoxia might suppress inflammatory p65/RelA at least partially via pre-induction of its antagonist IκBα by both mRNA induction and increased protein stability.

Addition of LPS strongly stimulated IκBα phosphorylation after 15 minutes followed by a decrease in total (and hence also phospho-) IκBα protein levels after 30 minutes. While the kinetics of IκBα phosphorylation appeared similar under normoxic and hypoxic conditions, the levels of total IκBα protein after 30 minutes were slightly higher under hypoxic conditions (Figure 8D), but the difference was not as pronounced as the increase in IκBα positive cell numbers at the same time point (Figure 8C), suggesting differential kinetics of the regulation of IκBα protein stability by LPS on the single cell level.

Interestingly, analysis of IKK phosphorylation, a surrogate marker for IKK-dependent IκBα kinase activity, revealed a much faster, transient IKK phosphorylation under hypoxic conditions (maximal at 15 minutes) than under normoxic conditions (maximal at 30 minutes). Lower IKK kinase activity after 30 minutes LPS stimulation under hypoxic conditions is consistent with increased IκBα levels, followed by attenuated proinflammatory gene expression.

## DISCUSSION

Mortality of colorectal cancer patients has previously been demonstrated to be associated with high HIF-1α, but not HIF-2α, mRNA and protein levels [24, 25]. The mouse model of colorectal cancer used in this study shows similar features: HIF-2α mRNA levels in MC-38 colon adenocarcinoma cells were at least two orders of magnitude lower than the HIF-1α mRNA levels. Moreover, no HIF-2α protein could be detected under hypoxic conditions (data not shown) and the hypoxic response of the canonical HIF target genes *Ca9* and *Phd3* was almost fully abrogated in shHIF-1α cells, demonstrating the lack of redundancy of the HIF response in these cells (supplementary Figure S3). This finding is in line with a recent report demonstrating that the *HIF2A*/*EPAS1* but not the *HIF1A* gene is epigenetically silenced in colonic adenocarcinoma specimens when compared with non-neoplastic tissue [26]. Therefore, shHIF-1α knock-down was sufficient to allow for the investigation of the role of the HIF signaling pathway in MC-38 cells.

NF-κB plays an important role during colorectal cancer progression and NF-κB has been shown to be a prognostic factor negatively associated with survival in patients with metastatic colorectal cancer [25, 27]. Colorectal cancer cells are likely to be exposed to bacterial LPS, derived from the gut microbiota, which is known to activate NF-κB via Toll-like receptor 4 (TLR4). Both TLR4 and its adapter MyD88 are increased in human sporadic colon cancer compared to surrounding normal and adenomatous epithelium, and are associated with poor patient prognosis [28], thereby implying a functional role for the LPS-TLR4-NF-κB pathway in colon cancer. NF-κB signaling was robustly activated in the epithelial MC-38 colon cancer cells used in this study in response to treatment with LPS, making them a suitable model to study the cross-talk between the hypoxic and the inflammatory pathways in colon cancer.

Although MC-38 cells responded to LPS with nuclear p65/RelA translocation as well as NF-κB target gene expression, no transcriptional induction of HIF-1α mRNA could be observed, as has previously reported for several other cellular systems [18-22]. Even under 3D culture conditions, where we generally observed a stronger inflammatory response as compared to 2D cultures, no upregulation of HIF-1α mRNA could be detected upon NF-κB activation (data not shown). Similarly, four additional human colon cancer cell lines (COLO-741, HT-29, SW-620 and CX-1) showed no HIF-1α mRNA upregulation following LPS treatment (data not shown). In contrast, we observed a 6-fold upregulation of HIF-1α mRNA in primary mouse peritoneal macrophages following LPS stimulation (supplementary Figure S4), strongly suggesting that transcriptional HIF-1α regulation by NF-κB is a cell type-specific phenomenon. However, the additional factors required to confer HIF-1α induction by NF-κB are currently unknown.

In contrast to several previous reports suggesting hypoxic NF-κB induction [11, 13, 15, 16], we found no increase in NF-κB activity upon acute (up to 4 hours) or chronic (up to 72 hours) hypoxic exposure of MC-38 cells. Of note, only four of 32 different cancer cell lines have been shown to sustainably upregulate NF-κB activity during a prolonged (48 hours) exposure to hypoxia, and this effect required the HPV-encoded E6 protein. Just a few HPV-negative cell lines showed a moderate and transient (up to 3 hours) induction of NF-κB activity upon exposure to hypoxia [14].

Regarding the absence of proinflammatory HIF-1α and hypoxic NF-κB induction in MC-38 colon cancer cells, we were surprised to find that under the same conditions, RNA deep sequencing revealed that 77% of the hypoxically induced genes required p65/RelA and still 18% of the inflammatory genes required HIF-1α. There are a number of genes known which have been reported to be regulated by both HIF-1 and NF-κB, including *NOS2* [29], *IL1B* [30, 31], *COX2* [16] and *PKM2* [32]. However, it is unlikely that HIF cooperates with NF-κB on virtually all of its target genes, and it seems more likely that epigenetic mechanisms might be the basis of this mutual cooperation [33, 34].

Rather than inducing NF-κB, hypoxia attenuated the proinflammatory response to LPS in MC-38 cells cultured under 2D and 3D conditions. RNA deep sequencing and RT-qPCR analysis of NF-κB target genes revealed only a minor role for HIF-1α in the decreased hypoxic induction of a few NF-κB target genes. A direct repressive HIF function is unlikely because genome-wide DNA association studies combined with transcriptional profiling and assessment of histone marks clearly revealed a transcriptional enhancer but no repressor function of both HIF-1α and HIF-2α [35].

Our findings are in line with two earlier studies performed using macrophages and synovial fibroblasts, respectively, which demonstrated that chemical hypoxia attenuated NF-κB dependent inflammatory genes induced by LPS but not by other proinflammatory stimuli [36, 37]. PHD oxygen sensing enzymes have been shown to be both positively and negatively implicated in NF-κB activation. PHD1 negatively regulates IκB kinase-β in HeLa cells, and PHD3 is a negative regulator of NF-κB in skeletal myoblast differentiation [13, 38]. On the other hand, LPS-induced cytokine expression in macrophages was suppressed by treatment with the PHD inhibitor dimethyloxalylglycine (DMOG) or siRNA-mediated knockdown of PHD1 and PHD2 [36]. DMOG pretreatment also attenuated the systemic LPS-induced activation of the NF-κB pathway and prevented endotoxic shock [39].

Quite unexpectedly, hypoxia inhibited the LPS-mediated nuclear translocation of p65/RelA in a HIF-independent manner. Apparently, hypoxia alone induced IκBα on both the mRNA and protein levels, and the activation of IKK kinase activity by LPS was of more transient nature under hypoxic than normoxic conditions, resulting in decreased IKK phosphorylation, slightly increased IκBα protein levels and clearly increased IκBα positive cell numbers after 30 minutes of LPS treatment. While these findings explain the attenuated inflammatory response, it still remains unclear as to how hypoxia attenuated IKK phosphorylation. Interestingly, it has recently been suggested that inhibition of the oxygen-dependent PHD1 and FIH protein hydroxylases blocks IL-1β signaling components of the TRAF6 complex involved in NF-κB activation [40]. Whether similar mechanisms are involved in the hypoxic attenuation of the LPS response needs to be investigated.

Both, the hypoxic and the inflammatory pathways are becoming increasingly well recognized as useful tools in cancer diagnosis as well as in tumor therapy [1]. Our results demonstrate a complex interplay between these two pathways in colon cancer cells. Whereas direct transcriptional cross-talks between HIF-1α and p65/RelA could not be confirmed in this cell type, strong indirect interactions between the two pathways could be demonstrated, suggesting that these two microenvironmental stimuli should always be considered simultaneously when designing novel approaches to tumor therapy.

## MATERIALS AND METHODS

### Cell culture

Mouse colon adenocarcinoma MC-38 GFP (if not indicated otherwise, referred to as “MC-38”) cells [41] were cultured in high glucose DMEM medium (Sigma, St. Louis, MO, USA; or Gibco, Carlsbad, CA, USA) supplemented with 10% FCS (Gibco), 50 IU/ml penicillin and 100 μg/ml streptomycin. MC-38 3D spheroid cultures were grown in hanging drops in 60-well microtest plates (Greiner, Frickenhausen, Germany) by seeding 2500 cells/25 μl drop 48 hours before the experiment. For hypoxia experiments, cells were grown in a gas-controlled workstation (InvivO_2_ 400, Ruskinn Technologies, Pencoed, UK). For inflammatory stimulation, MC-38 cells were treated with 1 μg/ml LPS derived from *E. coli* (Sigma). Before LPS treatment, 2D but not 3D cultures were starved overnight in 0.1% FCS.

### mRNA quantification

Total cellular RNA was extracted as described [42] or by using the Nucleospin RNA II extraction kit (Macherey-Nagel, Düren, Germany). cDNA was synthesized from 1-2 μg of total RNA using Affinity Script reverse transcriptase (RT) (Agilent Technologies, Santa Clara, CA, USA). Quantitative (q) PCR was performed using SybrGreen master mix (Sigma) on a MX3000P light cycler (Agilent Technologies). Primers were purchased from Microsynth (Balgach, Switzerland) and are listed in supplementary Table S2. RNA sample quality and equal inputs were assessed by RT-qPCR quantification of ribosomal protein S12 mRNA, and all data were expressed as ratios over S12 mRNA.

### Protein extraction and immunoblotting

Cells were washed twice with ice-cold PBS by centrifugation for 3 minutes at 1200 rpm. For total protein extraction, cells were resuspended in lysis buffer (10 mM Tris-HCl pH 8.0, 1 mM EDTA, 400 mM NaCl, 0.1% NP-40, freshly added 1 mM PMSF and Sigma's Protease Inhibitor Cocktail; Sigma's Phosphatase Inhibitor Cocktail was added for phospho-protein detection). For nuclear protein extraction, cells were resuspended in hypotonic buffer (10 mM HEPES/KOH pH 7.9, 10 mM KCl, 0.1 mM EDTA, 0.1 mM EGTA with freshly added 1 mM PMSF). After 15 minutes on ice, NP-40 was added to a final concentration of 0.1% and the samples were immediately vortexed for 20 seconds. Nuclei were pelleted for 30 seconds at 5000 rpm, washed with hypotonic buffer and extracted on ice in hypertonic extraction buffer (20 mM HEPES/KOH pH 7.9, 400 mM NaCl, 1mM EDTA, 1mM EGTA and protease inhibitors) for 10 minutes. Proteins were separated by 10% SDS-PAGE, electrotransferred to PVDF membranes and detected using anti-p65/RelA (Santa Cruz Biotechnology, Dallas, TX, USA), anti-HIF-1α (Novus Biologicals, Littletone, CO, USA), anti-HDAC1 (Abcam, Cambridge, UK), anti-SP1 (Santa Cruz Biotechnology), anti-phospho-Ser176/180-IKKα/β anti-IKKβ, anti-phospho-Ser32/36-IκBα and anti-IκBα (all from Cell Signaling Technology, Danvers, MA, USA), or anti-β-actin (Sigma) antibodies.

### Immunofluorescence microscopy

Following fixation with 4% paraformaldehyde for 30 minutes at room temperature, MC-38 cells were washed twice with PBS, permeabilized with 0.1% saponin, free aldehyde groups quenched with 20 mM glycine, and unspecific binding sites blocked with 10% FCS for 30 minutes. Proteins were detected by incubation for 1 hour at 37°C with anti-p65/RelA (Santa Cruz Biotechnology) or anti-IκBα (Cell Signaling Technology) antibodies. After washing with PBS, the respective secondary antibodies labeled with Cy3 (Jackson Immunoresearch, West Grove, PA, USA), Alexa488 or Alexa568 (Molecular Probes, Carlsbad, CA, USA) were applied, and nuclei were counter-stained with DAPI. Samples were mounted using Mowiol (Millipore, Darmstadt, Germany) and analyzed by fluorescence microscopy. Spheroids were fixed in 10% formalin for 30 minutes at room temperature and processed for histological analysis using previously described techniques [43]. Dewaxed paraffin sections (5 μm) were rehydrated, blocked with 10% normal goat serum and processed as described above.

### RNA interference

Knock-down of specific mRNAs was achieved by lentiviral transduction of short hairpin RNA (shRNA) vectors driven by the U6 promoter in the pLKO.1-puro plasmid (Sigma). Vectors targeting mouse HIF-1α (shHIF-1α) and non-target controls (shMOCK_A) were purchased from Sigma. To construct the shp65 vector, the oligonucleotides 5′-gatcccca gggcaaactgtagagtcattcaagagatgactctacagtttgcccttttttggaaa-3′ and 5′-agcttttcc aaaaagggcaaactgtagagtcatctcttgaatga ctctacagtttgccctggg-3′ were annealed, phosphorylated and ligated into the pSUPER vector. A BamHI/SalI fragment of the H1-RNA promoter was ligated into the pRDI292 vector (kind gift from P. O. Hassa, Zurich, Switzerland) and used for expression of shp65.

### RNA sequencing and data deposition

RNA was extracted and pooled from 3 independent 3D-cultures grown under hypoxic or normoxic conditions. Ribosomal RNA in the pooled RNA was depleted using Encore Complete RNA-Seq Library Systems (NuGEN, San Carlos, CA, USA) and RNA was sequenced using an Illumina HiSeq 2000 sequencer (San Diego, CA, USA) at the Functional Genomics Center Zurich. Isoform and gene expression levels were computed with RSEM (Version 1.2.0; http://www.biomedcentral.com/1471-2105/12/323). RSEM was run in stranded mode with the additional option to estimate the distribution of the read start positions. The expression levels (RSEM's posterior estimate of the read counts per transcript normalized for gene length) were normalized across samples using the geometric mean of all expression signals. To attenuate expression ratios for low abundance genes, a fixed value of 5 was added to all expression values before computing the log-ratio. Threshold levels for hypoxia- or LPS-dependent up- or down-regulation were set at >1.5 or <0.67, respectively, as were the threshold levels for HIF-1α or p65/RelA dependency of up- or down-regulated gene expression when compared to the respective shMOCK controls. Only genes with a minimal expression level of 10 were considered biologically relevant. All data were deposited in the Short Read Archive (SRA) of the NCBI and are accessible through the ID SRP043151.

### Statistical analysis

Unless indicated otherwise, all experiments were repeated at least three times independently. Data are shown as mean values + SD. Student's t-tests or two-way ANOVA were applied to analyze the data as indicated, with a *p*-value < 0.05 considered to be statistically significant.

## SUPPLEMENTARY FIGURES AND TABLES



## References

[1] Eltzschig HK, Carmeliet P (2011). Hypoxia and inflammation. N Engl J Med.

[2] Kaelin WG, Ratcliffe PJ (2008). Oxygen sensing by metazoans: the central role of the HIF hydroxylase pathway. Mol Cell.

[3] Semenza GL (2010). Defining the role of hypoxia-inducible factor 1 in cancer biology and therapeutics. Oncogene.

[4] Wenger RH, Stiehl DP, Camenisch G (2005). Integration of oxygen signaling at the consensus HRE. Sci STKE.

[5] Pouysségur J, Dayan F, Mazure NM (2006). Hypoxia signalling in cancer and approaches to enforce tumour regression. Nature.

[6] Bernstein CN, Blanchard JF, Kliewer E, Wajda A (2001). Cancer risk in patients with inflammatory bowel disease: a population-based study. Cancer.

[7] Mantovani A, Allavena P, Sica A, Balkwill F (2008). Cancer-related inflammation. Nature.

[8] Murdoch C, Muthana M, Coffelt SB, Lewis CE (2008). The role of myeloid cells in the promotion of tumour angiogenesis. Nat Rev Cancer.

[9] Mercurio F, Zhu H, Murray BW, Shevchenko A, Bennett BL, Li J, Young DB, Barbosa M, Mann M, Manning A, Rao A (1997). IKK-1 and IKK-2: cytokine-activated IκB kinases essential for NF-κB activation. Science.

[10] Dolcet X, Llobet D, Pallares J, Matias-Guiu X (2005). NF-κB in development and progression of human cancer. Virchows Arch.

[11] Hirani N, Antonicelli F, Strieter RM, Wiesener MS, Ratcliffe PJ, Haslett C, Donnelly SC (2001). The regulation of interleukin-8 by hypoxia in human macrophages - a potential role in the pathogenesis of the acute respiratory distress syndrome (ARDS). Mol Med.

[12] Walmsley SR, Print C, Farahi N, Peyssonnaux C, Johnson RS, Cramer T, Sobolewski A, Condliffe AM, Cowburn AS, Johnson N, Chilvers ER (2005). Hypoxia-induced neutrophil survival is mediated by HIF-1α-dependent NF-κB activity. J Exp Med.

[13] Cummins EP, Berra E, Comerford KM, Ginouves A, Fitzgerald KT, Seeballuck F, Godson C, Nielsen JE, Moynagh P, Pouysségur J, Taylor CT (2006). Prolyl hydroxylase-1 negatively regulates IκB kinase-β, giving insight into hypoxia-induced NFκB activity. Proc Natl Acad Sci USA.

[14] An J, Mo D, Liu H, Veena MS, Srivatsan ES, Massoumi R, Rettig MB (2008). Inactivation of the CYLD deubiquitinase by HPV E6 mediates hypoxia-induced NF-κB activation. Cancer Cell.

[15] Culver C, Sundqvist A, Mudie S, Melvin A, Xirodimas D, Rocha S (2010). Mechanism of hypoxia-induced NF-κB. Mol Cell Biol.

[16] Fitzpatrick SF, Tambuwala MM, Bruning U, Schaible B, Scholz CC, Byrne A, O'Connor A, Gallagher WM, Lenihan CR, Garvey JF, Howell K, Fallon PG, Cummins EP (2011). An intact canonical NF-κB pathway is required for inflammatory gene expression in response to hypoxia. J Immunol.

[17] Cockman ME, Lancaster DE, Stolze IP, Hewitson KS, McDonough MA, Coleman ML, Coles CH, Yu X, Hay RT, Ley SC, Pugh CW, Oldham NJ, Masson N (2006). Posttranslational hydroxylation of ankyrin repeats in IκB proteins by the hypoxia-inducible factor (HIF) asparaginyl hydroxylase, factor inhibiting HIF (FIH). Proc Natl Acad Sci USA.

[18] Belaiba RS, Bonello S, Zähringer C, Schmidt S, Hess J, Kietzmann T, Görlach A (2007). Hypoxia up-regulates hypoxia-inducible factor-1α transcription by involving phosphatidylinositol 3-kinase and nuclear factor kB in pulmonary artery smooth muscle cells. Mol Biol Cell.

[19] Bonello S, Zahringer C, BelAiba RS, Djordjevic T, Hess J, Michiels C, Kietzmann T, Görlach A (2007). Reactive oxygen species activate the HIF-1α promoter via a functional NFkB site. Arterioscler Thromb Vasc Biol.

[20] Rius J, Guma M, Schachtrup C, Akassoglou K, Zinkernagel AS, Nizet V, Johnson RS, Haddad GG, Karin M (2008). NF-κB links innate immunity to the hypoxic response through transcriptional regulation of HIF-1α. Nature.

[21] van Uden P, Kenneth NS, Rocha S (2008). Regulation of hypoxia-inducible factor-1a by NF-κB. Biochem J.

[22] Yoshida T, Hashimura M, Mastumoto T, Tazo Y, Inoue H, Kuwata T, Saegusa M (2013). Transcriptional upregulation of HIF-1α by NF-κB/p65 and its associations with β-catenin/p300 complexes in endometrial carcinoma cells. Lab Invest.

[23] Gassmann M, Fandrey J, Bichet S, Wartenberg M, Marti HH, Bauer C, Wenger RH, Acker H (1996). Oxygen supply and oxygen-dependent gene expression in differentiating embryonic stem cells. Proc Natl Acad Sci USA.

[24] Baba Y, Nosho K, Shima K, Irahara N, Chan AT, Meyerhardt JA, Chung DC, Giovannucci EL, Fuchs CS, Ogino S (2010). HIF1A overexpression is associated with poor prognosis in a cohort of 731 colorectal cancers. Am J Pathol.

[25] Novell A, Martinez-Alonso M, Mira M, Tarragona J, Salud A, Matias-Guiu X (2014). Prognostic value of c-FLIPL/s, HIF-1α, and NF-κB in stage II and III rectal cancer. Virchows Arch.

[26] Rawluszko-Wieczorek AA, Horbacka K, Krokowicz P, Misztal M, Jagodzinski PP (2014). Prognostic potential of DNA methylation and transcript levels of HIF1A and EPAS1 in colorectal cancer. Mol Cancer Res.

[27] Puvvada SD, Funkhouser WK, Greene K, Deal A, Chu H, Baldwin AS, Tepper JE, O'Neil BH (2010). NF-κB and Bcl-3 activation are prognostic in metastatic colorectal cancer. Oncology.

[28] Wang EL, Qian ZR, Nakasono M, Tanahashi T, Yoshimoto K, Bando Y, Kudo E, Shimada M, Sano T (2010). High expression of Toll-like receptor 4/myeloid differentiation factor 88 signals correlates with poor prognosis in colorectal cancer. Br J Cancer.

[29] Melillo G, Musso T, Sica A, Taylor LS, Cox GW, Varesio L (1995). A hypoxia-responsive element mediates a novel pathway of activation of the inducible nitric oxide synthase promoter. J Exp Med.

[30] Zhang W, Petrovic JM, Callaghan D, Jones A, Cui H, Howlett C, Stanimirovic D (2006). Evidence that hypoxia-inducible factor-1 (HIF-1) mediates transcriptional activation of interleukin-1β (IL-1β) in astrocyte cultures. J Neuroimmunol.

[31] Tannahill GM, Curtis AM, Adamik J, Palsson-McDermott EM, McGettrick AF, Goel G, Frezza C, Bernard NJ, Kelly B, Foley NH, Zheng L, Gardet A, Tong Z (2013). Succinate is an inflammatory signal that induces IL-1β through HIF-1α. Nature.

[32] Xu Q, Liu LZ, Yin Y, He J, Li Q, Qian X, You Y, Lu Z, Peiper SC, Shu Y, Jiang BH (2015). Regulatory circuit of PKM2/NF-κB/miR-148a/152-modulated tumor angiogenesis and cancer progression. Oncogene.

[33] Brigati C, Banelli B, di Vinci A, Casciano I, Allemanni G, Forlani A, Borzi L, Romani M (2010). Inflammation, HIF-1, and the epigenetics that follows. Mediators Inflamm.

[34] Tsai YP, Wu KJ (2014). Epigenetic regulation of hypoxia-responsive gene expression: focusing on chromatin and DNA modifications. Int J Cancer.

[35] Schödel J, Oikonomopoulos S, Ragoussis J, Pugh CW, Ratcliffe PJ, Mole DR (2011). High-resolution genome-wide mapping of HIF-binding sites by ChIP-seq. Blood.

[36] Takeda K, Ichiki T, Narabayashi E, Inanaga K, Miyazaki R, Hashimoto T, Matsuura H, Ikeda J, Miyata T, Sunagawa K (2009). Inhibition of prolyl hydroxylase domain-containing protein suppressed lipopolysaccharide-induced TNF-α expression. Arterioscler Thromb Vasc Biol.

[37] Hu F, Mu R, Zhu J, Shi L, Li Y, Liu X, Shao W, Li G, Li M, Su Y, Cohen PL, Qiu X, Li Z (2014). Hypoxia and hypoxia-inducible factor-1α provoke toll-like receptor signalling-induced inflammation in rheumatoid arthritis. Ann Rheum Dis.

[38] Fu J, Taubman MB (2010). Prolyl hydroxylase EGLN3 regulates skeletal myoblast differentiation through an NF-κB-dependent pathway. J Biol Chem.

[39] Hams E, Saunders SP, Cummins EP, O'Connor A, Tambuwala MT, Gallagher WM, Byrne A, Campos-Torres A, Moynagh PM, Jobin C, Taylor CT, Fallon PG (2011). The hydroxylase inhibitor dimethyloxallyl glycine attenuates endotoxic shock via alternative activation of macrophages and IL-10 production by B1 cells. Shock.

[40] Scholz CC, Cavadas MA, Tambuwala MM, Hams E, Rodriguez J, von Kriegsheim A, Cotter P, Bruning U, Fallon PG, Cheong A, Cummins EP, Taylor CT (2013). Regulation of IL-1β-induced NF-κB by hydroxylases links key hypoxic and inflammatory signaling pathways. Proc Natl Acad Sci USA.

[41] Borsig L, Wong R, Hynes RO, Varki NM, Varki A (2002). Synergistic effects of L- and P-selectin in facilitating tumor metastasis can involve non-mucin ligands and implicate leukocytes as enhancers of metastasis. Proc Natl Acad Sci USA.

[42] Chomczynski P, Sacchi N (1987). Single-step method of RNA isolation by acid guanidinium thiocyanate-phenol-chloroform extraction. Anal Biochem.

[43] Mirsaidi A, Tiaden AN, Richards PJ (2013). Preparation and osteogenic differentiation of scaffold-free mouse adipose-derived stromal cell microtissue spheroids (ASC-MT). Current protocols in stem cell biology.

