# Supplementary material for: Hypoxia attenuates the proinflammatory response in colon cancer cells by regulating IκB
Source: Oncotarget. 2015 Apr 29;6(24):20288–301. doi: 10.18632/oncotarget.3961 (PMC4653005; doi:10.18632/oncotarget.3961)
Supplement: Supplementary file 1 [file oncotarget-06-20288-s001.pdf]

Hypoxia attenuates the proinflammatory response in colon cancer cells by regulating IκB

SUPPLEMENTARY MATERIAL

Table S1: Gene regulation revealed by RNA sequencing.

**Conditions**	**+Hyp/-LPS vs. -Hyp/-LPS**	**+Hyp/+LPS vs. -Hyp/-LPS**	**-Hyp/+LPS vs. -Hyp/-LPS**	**+Hyp/+LPS vs. +Hyp/-LPS**
upregulated genes (total)	924	645	277	141
regulated by:				
HIF-1α (+)	663	393	45	26
HIF-1α (-)	1	7	6	9
HIF-1α (total)	664	400	51	35
p65/RelA (+)	708	135	164	34
p65/RelA (-)	2	4	0	4
p65/RelA (total)	710	139	164	38
HIF-1α (+), p65/RelA (+)	538	77	31	11
HIF-1α (-), p65/RelA (-)	0	0	0	1
HIF-1α (+), p65/RelA (-)	1	4	0	0
HIF-1α (-), p65/RelA (+)	0	4	2	5
HIF-1α and p65/RelA (total)	539	85	33	17
downregulated genes (total)	429	332	36	105
regulated by:				
HIF-1α (+)	12	12	0	2
HIF-1α (-)	106	78	17	53
HIF-1α (total)	118	90	17	55
p65/RelA (+)	155	10	0	2
p65/RelA (-)	50	60	21	76
p65/RelA (total)	205	70	21	78
HIF-1α (+), p65/RelA (+)	6	3	0	0
HIF-1α (-), p65/RelA (-)	16	35	9	36
HIF-1α (+), p65/RelA (-)	0	0	0	2
HIF-1α (-), p65/RelA (+)	32	0	0	1
HIF-1α and p65/RelA (total)	54	38	9	39

Table S2: Primers used for real-time PCR.

mRNA	forward primer	reverse primer
CAIX<br>carbonic anhydrase 9	5’-gctgtcccatttggaagaaa-3’	5’-ggaaggaagcctcaatcgtt-3’
CCL20<br>chemokine (C-C motif) ligand 20	5’-cgactgttgcctctcgtaca-3’	5’-gaggaggttcacagccctttt-3’
COX-2<br>cytochrome oxidase 2	5’-tcctcctggaacatggactc-3’	5’-ccccaaagatagcatctgga-3’
CSF2<br>colony stimulating factor 2	5’-acatgcctgtcacgttgaat-3’	5’-ttgagtttggtgaaattgcc-3’
CXCL5<br>chemokine (C-X-C motif) ligand 5	5’-gctgctgtgtcatgcagaaaccta-3’	5’-gacattatgccatactacgaagacatc-3’
GLUT-1<br>glucose transporter 1	5’-tctctgtcggcctctttgtt-3’	5’-gcagaagggcaacaggatac-3’
HIF-1α<br>hypoxia-inducible factor 1α	5’-acaagtcaccacaggacag-3’	5’-agggagaaaatcaagtcg-3’
HIF-2α<br>hypoxia-inducible factor 2α	5’-taaagcggcagctggagtat-3’	5’-actgggaggcatagcactgt-3’
IκBα<br>Inhibitor of NF-κB α	5'-caactacaatggccacacg-3'	5'-caggattctgcaggtccac-3'
IL-6<br>interleukin 6	5’-tcctctctgcaagagacttccatcc-3’	5’-aagcctccgacttgtgaagtggt-3’
p65/RelA<br>NF-κB subunit p65	5’-gcgtacacattctggggagt-3’	5’-accgaagcaggagctatcaa-3’
PHD3<br>prolyl-4-hydroxylase domain protein 3	5’-caacttcctcctgtccctca-3’	5’-ggctggacttcatgtggatt-3’
S12<br>ribosomal protein S12	5’-gaagctgccaaagccttaga-3’	5’-aactgcaaccaaccaccttc-3’
TNF-α<br>tumor necrosis factor α	5’-gtcgtagcaaaccaccaagtgg-3’	5’-gagatagcaaatcggctgacgg-3’

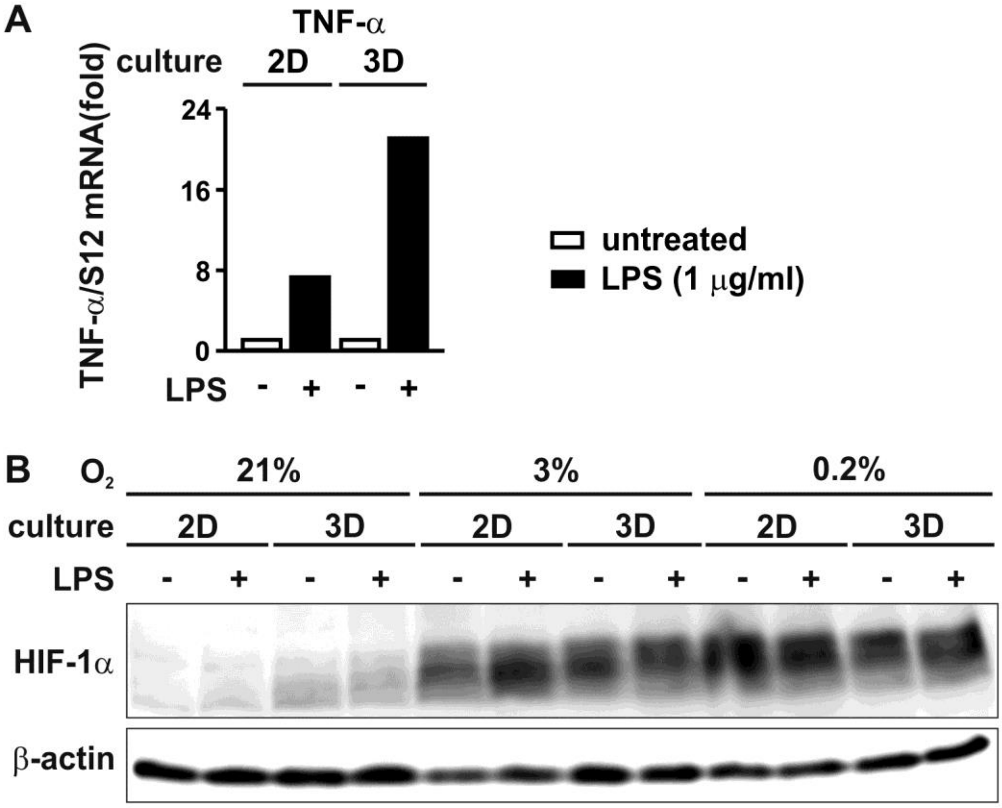

Figure S1: Spheroid cultures of MC-38 colon carcinoma cells. MC-38 cell were either cultured as sub-confluent two-dimensional monolayers in tissue culture plates ("2D") or as three-dimensional spheroids in hanging drops ("3D") for 48 hours before the experiment was started. **A.** Both 2D and 3D cultures were stimulated with 1 μg/ml LPS for 1 hour and the TNF-α mRNA levels were determined by RT-qPCR. **B**. Cells were exposed to hypoxia (3% or 0.2% O_2) for 8 hours and/or 1 μg/ml LPS for the last 2 hours before harvesting. HIF-1α protein was detected by immunoblotting of 50 μg of total protein extracts. β-Actin served as loading control.

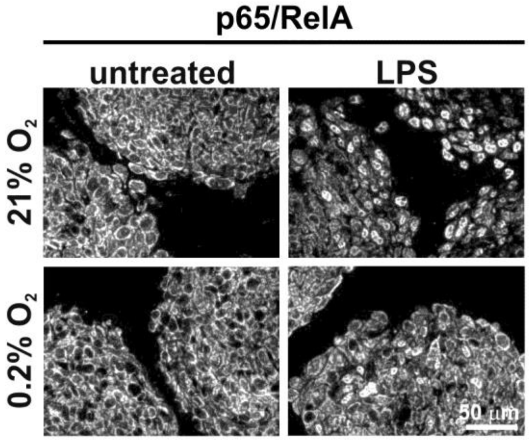

Figure S2: Nuclear translocation of p65/RelA in hypoxic spheroid cultures. Immunofluorescence microscopy of p65/RelA in MC-38 cells grown as spheroids in hanging drop cultures for 48 hours before exposure to hypoxia (0.2% oxygen) for 8 hours and/or 1 μg/ml LPS for the last 40 minutes before harvesting.

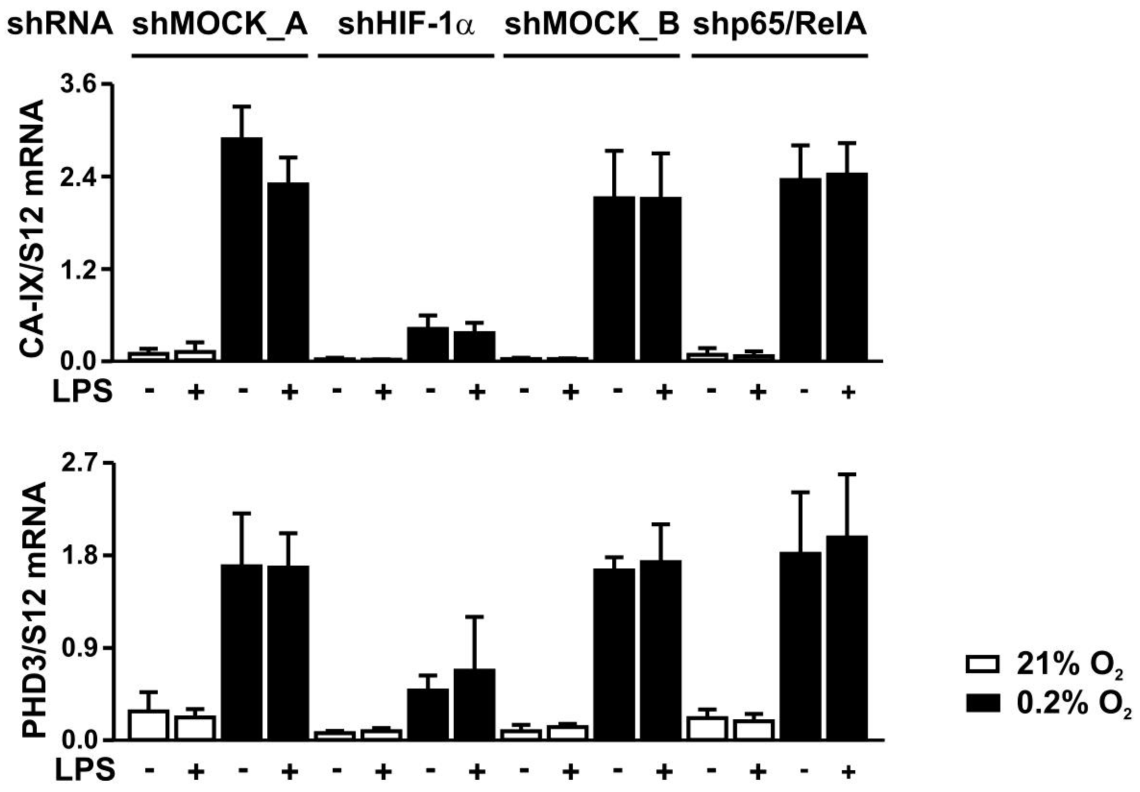

Figure S3: CAIX and PHD3 mRNA levels in shHIF-1α MC-38 cells. Hypoxic induction of the canonical HIF target genes *Ca9* and *Phd3* was quantified by RT-qPCR. Shown are mean values + SD of mRNA ratios relative to the constitutively expressed ribosomal protein S12 mRNA levels of n = 3 to 4 independent experiments.

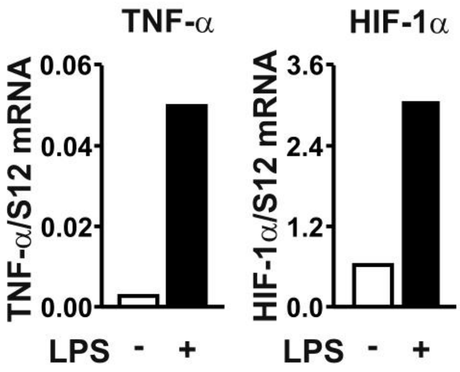

Figure S4: Inflammatory response of macrophages. Transcript levels of TNF-α and HIF-1α were determined by RT-qPCR in mouse primary peritoneal macrophages following stimulation with 100 ng/ml LPS for 8 hours.
